# Case Report: A Rare Case of an Obstructed Floating Kidney Incarcerated Within a Massive Scrotal Hernia: Management at a Norwegian Hospital by Surgeons With Experience in Africa

**DOI:** 10.3389/jaws.2025.13914

**Published:** 2025-05-15

**Authors:** Robbert-Jan Lindeman, Chris Oppong, Odd Mjåland

**Affiliations:** ^1^ Gastrointestinal Surgery Department, Stavanger University Hospital, Stavanger, Norway; ^2^ General Surgery Department, Plymouth Nuffield Hospital, Plymouth, United Kingdom; ^3^ Research Department, Sorlandet Hospital, Kristiansand, Norway

**Keywords:** inguinal hernia, massive inguinoscrotal hernia, obstructed kidney, surgical technique, global surgery

## Abstract

Open mesh repair for inguinal hernia is one of the most commonly performed surgical procedures worldwide. Pediatric and symptomatic inguinal hernias are preferably treated at an early stage, according to current guidelines. Consequently, massive inguinoscrotal hernias are rarely seen in most high-income countries. In contrast, scrotal hernia repair account for 67% of all inguinal hernia repairs in low-resource countries. Recently, there has been an increased focus on scrotal hernias as a specific type of pathology. Alterations of anatomical landmarks and disruption of anatomical layers make these hernias a surgical challenge, requiring a different surgical approach. Due to the rarity in high resource countries, most general surgeons have limited experience in their surgical management. In this case report we present a challenging acute case of massive inguinoscrotal hernia with incarceration of the right kidney. The authors of this paper are part of the Norwegian-British team for Operation Hernia, a UK-based humanitarian Trust with 18 years of experience in hernia surgery in low resource countries. Through the years, the team has gathered extensive experience in the management of large inguinoscrotal hernias. This paper describes the management of a complex case, which was treated with the combined experience of frugal surgery on Operation Hernia missions and the almost limitless resources in a Norwegian hospital.

## Introduction

Inguinal hernia is one of the most common surgical procedures performed worldwide. Open mesh repair has been the golden standard for half a century. However, laparoscopic approaches such as TEP (Totally Extraperitoneal) and TAPP (Transabdominal Preperitoneal) have gradually become the preferred technique in most high-income countries due to slightly faster recovery and reduced chronic pain [1]. In these healthcare systems, surgical capacity is high and matches the incidence of hernias. Hence, symptomatic hernias are treated at an early stage and scrotal hernias represent only 6% of all operated cases [2]. As a result, experience with complex large inguinoscrotal hernias is limited among most general surgeons.

The opposite is true for low and middle-income countries (LMIC), where up to 67% of all inguinal hernia case are scrotal hernias [2]. The operation rate for inguinal hernia is generally low, resulting in a large pool of untreated patients with longstanding, complex inguinoscrotal hernias [3]. Due to this, the senior doctors who perform hernia surgery in the LMIC have a relatively high level of competence in the treatment of complex inguinoscrotal hernias.

Recently, there has been more focus on scrotal hernias as a specific type of pathology [2, 4, 5]. Alterations of anatomical landmarks and disruption of anatomical layers make large scrotal hernias a challenging entity, demanding different surgical approach. In this paper we present the management of a rare case of massive inguinoscrotal hernia with renal incarceration.

Two of the authors of this paper are part of the Norwegian Operation Hernia Team, the third is a founder of the Operation Hernia Trust. Having accumulated experience through multiple missions over several years in Ghana, the Norwegian team was challenged with this complex caser. The aim of this paper is to describe surgical decision-making which was informed by he experience from work in a low resource environment and the almost limitless resources in a Norwegian hospital.

## Case Description

**FIGURE 1:**
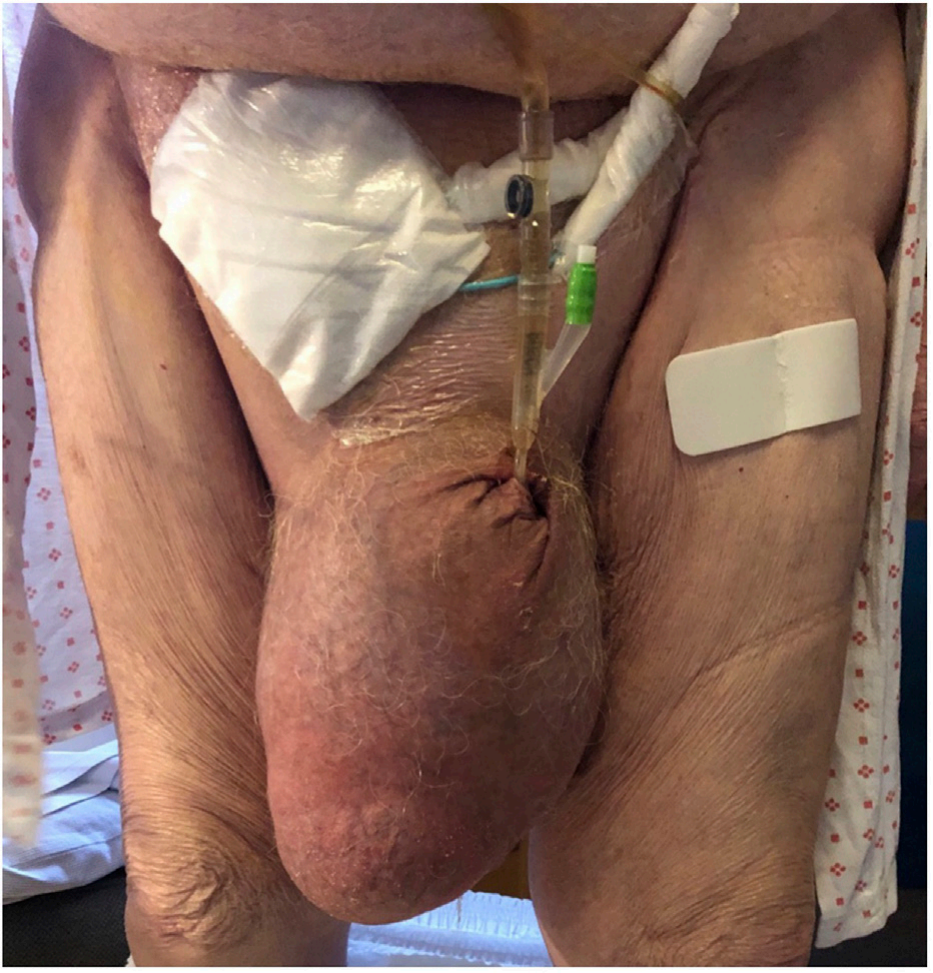
Massive inguinoscrotal hernia with kidney involvement.

The patient is an 83-year-old Caucasian obese male (BMI 39) with and hypertension. He underwent acute open mesh repair for an incarcerated umbilical hernia 10 years ago. The patient was admitted to the acute ward with a massive irreducible scrotal hernia, which descended to his right knee (S3 according to EHS classification) (Figure 1) [2]. He was septic, in acute renal failure and in severe pain. On clinical examination he was tachycardic, febrile and hypotensive. The scrotal skin was edematous and excoriated in places due to leakage of urine from the retracted penis.

### Investigations

Blood samples revealed: CRP: 240 mg/L, Leu: 28.1 x10E9/L, GFR: 29 mL/min/1.73 m^2^ (75), Urea: 18 mmol/L, Creatinine: 246 μmol/L, Hb 11.2 g/dL, Na 135 mmol/L, K: 3.8 mmol/L.

## Timeline

The patient had developed a large inguinoscrotal hernia over 20 years. Two years before the acute admission he was referred by his general practitioner due to “the size of the hernia.” Together with the patient it was decided not to operate. The case was never discussed at a dedicated abdominal wall meeting. Due to increasing symptoms the patient was put on a waiting list for surgery but was acutely admitted with sepsis and incarceration 4 weeks later.

## Diagnostic Assessment

### CT-Scan

**FIGURE 2:**
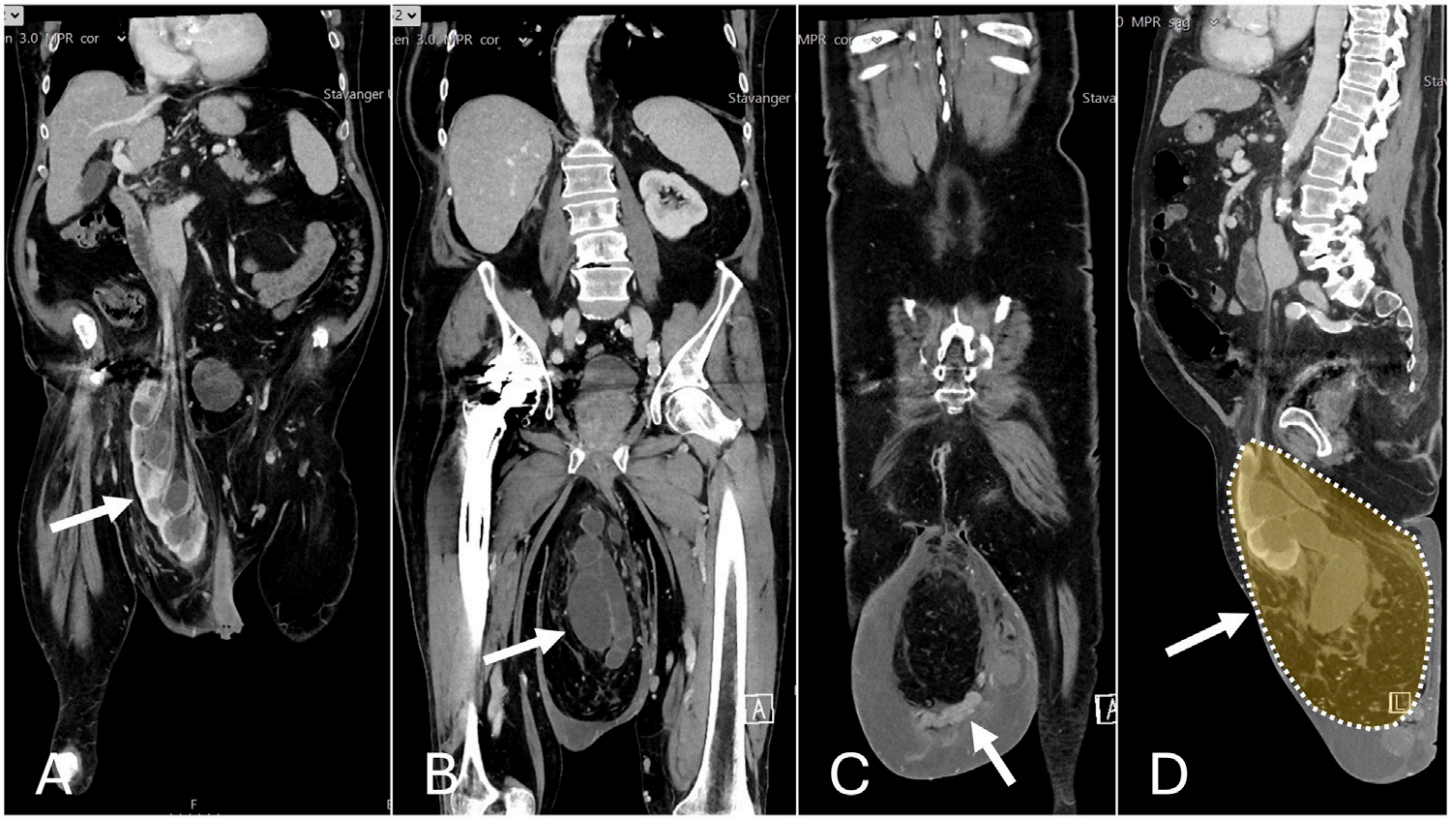
Preoperative CT scan. **(A)** Incarcerated kidney. **(B)** Hydroureter. **(C)** Varicose veins and edema. **(D)** Loss of domain.

- Massive inguinoscrotal hernia with incarcerated kidney with hydronephrosis, severe hydroureter and an elongated vascular supply measuring 40 cm (Figure 2).
- Approximately 20% loss of domain.
- Varicose veins in the spermatic cord with proximal vein thrombosis due to external pressure of the incarcerated kidney.
- The renal vessels arise from the aorta in their usual position just below the superior mesenteric artery.
- Double-collecting system of the right kidney.
- Large amount of edema into the subcutaneous.
- The left kidney was located in its normal anatomical position.

## Therapeutic Intervention

### Acute Management

Upon emergency admission, sepsis treatment was initiated. Two percutaneous pigtail drains were inserted into the double collecting system of the right kidney. The patient recovered from sepsis and was discharged to a rehabilitation home after 6 days. A renography performed 4 weeks later revealed equal distribution of kidney function. This emphasized the need for preservation of the kidney during definitive surgical treatment.

### Treatment Plan

A treatment plan was established and discussed with experts from Operation Hernia and the European Hernia Society (EHS). However, risk assessment and patient information was challenging, as data regarding morbidity and mortality in massive inguinoscrotal hernia is lacking. Several treatment options were discussed:

1. No Surgery. Leave the external drainage in place and change the pyelostomies every third month. Initially, this was the patient´s preferred choice. After consulting his family and further considerations, the patient changed his mind.
2. Stoppa procedure. In view of the patient’s comorbidity and frailty it was considered inappropriate. Increased tissue trauma and operating time favoured a different approach. Moreover, the surgical team did not have enough experience with the Stoppa procedure. The patient would have had to be referred to another hospital, something the patient did not want.
3. Lichtenstein repair. From 2019 to 2023, the Norwegian team successfully treated over 75 cases using the Lichtenstein procedure for large scrotal hernias, where the sac extends beyond the inner thigh, in a rural region of Southern Ghana, without expanding the volume of the abdominal cavity.

Considering the team’s experience and their own available data, it was reasonable to expect favorable outcomes with acceptable risks. Therefore, a Lichtenstein repair was recommended. However, given the patient’s significant deterioration in general health, along with the nature of the planned operation, it was essential to implement a prehabilitation program [6].

### Prehabilitation

The patient was admitted to a rehabilitation center for preoperative optimization 8 weeks prior to surgery. He followed a personalized training program supervised by a physiotherapist two to three times a week. On other days, he performed muscle strengthening exercises. During admission the patient was consulted by a dietitian that prescribed protein supplements to correct the patient’s hypoalbuminemia. The patient received blood transfusion for optimization of the hemoglobin levels during an acute admission for stress-related bleeding peptic ulcer 6 weeks prior to surgery. PPI (Proton Pump Inhibitor) was started, and the treatment was continued with a maintenance dose. Two weeks later, the patient was admitted to the hospital for 4 days due to a COVID-19 infection complicated by pneumonia.

Standardized functional tests (SPPB, frailty score, TUG, 6-minute walk test, and grip strength) were conducted approximately eight and one week before surgery, as well as six weeks and six months after surgery. The tests revealed substantial increase in muscle strength and better body mobility, both prior and after surgery. On the day of surgery albumin and hemoglobin levels were in the normal range, as were kidney function tests and electrolytes.

### Botulinum Toxin a (Botox) Injections

The estimated 20% loss of domain necessitated augmentation of the abdominal cavity. Four weeks preoperatively, 100 units of Botulinum toxin (Xeomin) were injected under ultrasound-guidance into the oblique muscles on either side of the abdomen. We used a 3-point technique, where each injection contained 32 units of botox diluted within 16 mL NaCl.

### Surgical Procedure

Step 1: Preparations

General anesthesia. Informed consent, including orchidectomy. Patient received prophylactic antibiotics. Both pyelostomies were removed and a urine catheter was placed before skin sterilization. A first attempt to reduce the hernia under general anesthesia was unsuccessful.

Step 2: Access to inguinal canal

**FIGURE 3:**
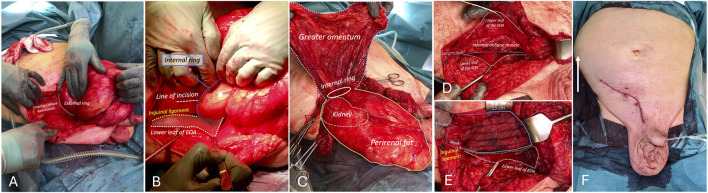
Procedural steps. **(A)**. Enlarged EOA. **(B)** Line of incision of enlarged internal ring. **(C)** Sliding hernia containing retroperitoneum with kidney. Perirenal fat and hydroureter. Anteriorly, the true sac containing the greater omentum. **(D)** Reconstruction of posterior wall. **(E)** Mesh placement. **(F)** Bulging of right flank after reduction due to preoperative botox injections (white arrow).

The skin incision was extended to include the scrotal skin [4]. The hernia contents were dissected out of the scrotum using mainly blunt dissection and a second unsuccessful attempt at reduction was made. The grossly stretched external oblique aponeurosis (EOA) was incised (Figure 3A). A third attempt at reduction was unsuccessful.

Step 3: Dissection of the hernia sac

A large multi-layered hernia sac with extensive adhesions was found. The dissection started with identifying the different layers of the hernia contents. A large mass protruding from the internal ring was identified (Figure 3B). Initially, we thought this was the hernia sac. In an attempt to open the sac, a largely distended ureter together with large amounts of perirenal fat were identified. At this point it became clear that we dealt with a sliding hernia consisting of the retroperitoneal kidney and Gerota´s fascia. The true hernia sac was then identified anterior to the kidney containing the greater omentum (Figure 3B). The defect in the retroperitoneum was closed before reduction.

Step 4: Orchidectomy

To enable reduction, a right-sided orchiectomy was performed. The cord was densely adherent to the retroperitoneal tissues. The testicular venous plexus was grossly dilated due to proximal vein thrombosis and bled easily.

Step 5: Lateral incision of the internal ring

To widen the internal ring, the internal oblique muscle was carefully divided laterally on to a blade handle. Finally, a successful reduction was performed (Figure 3C). The patient was hemodynamically stable, and ventilation was not adversely affected during reduction due to good laxity of the abdominal wall because of preoperative botox treatment (Figure 3F).

Step 6: Reconstruction of the inguinal canal

The peritoneal hernia sac containing the greater omentum was completely distorted by the extensive adhesion formation and was not identified as a distinct structure. However, the internal ring was clearly identified and closed completely after orchidectomy. The posterior wall of the inguinal canal was reconstructed by suturing the conjoint tendon without tension to the inguinal ligament with a poly-p-dioxanone (PDS) running suture (Figure 3D).

Step 7: Mesh placement

A 10 × 15 cm polypropylene mesh was implanted and fixated towards the rectus sheath overlying the pubic tubercle and along the inguinal ligament with a non-absorbable running suture (Figure 3E).

Step 8: closure

The stretched EOA was trimmed and closed over the mesh. The wound was closed in layers. No drain was placed. A scrotal compression bandage was applied for 24 h. Oral antibiotics were continued for 5 days.

### Follow-Up and Outcomes

**FIGURE 4:**
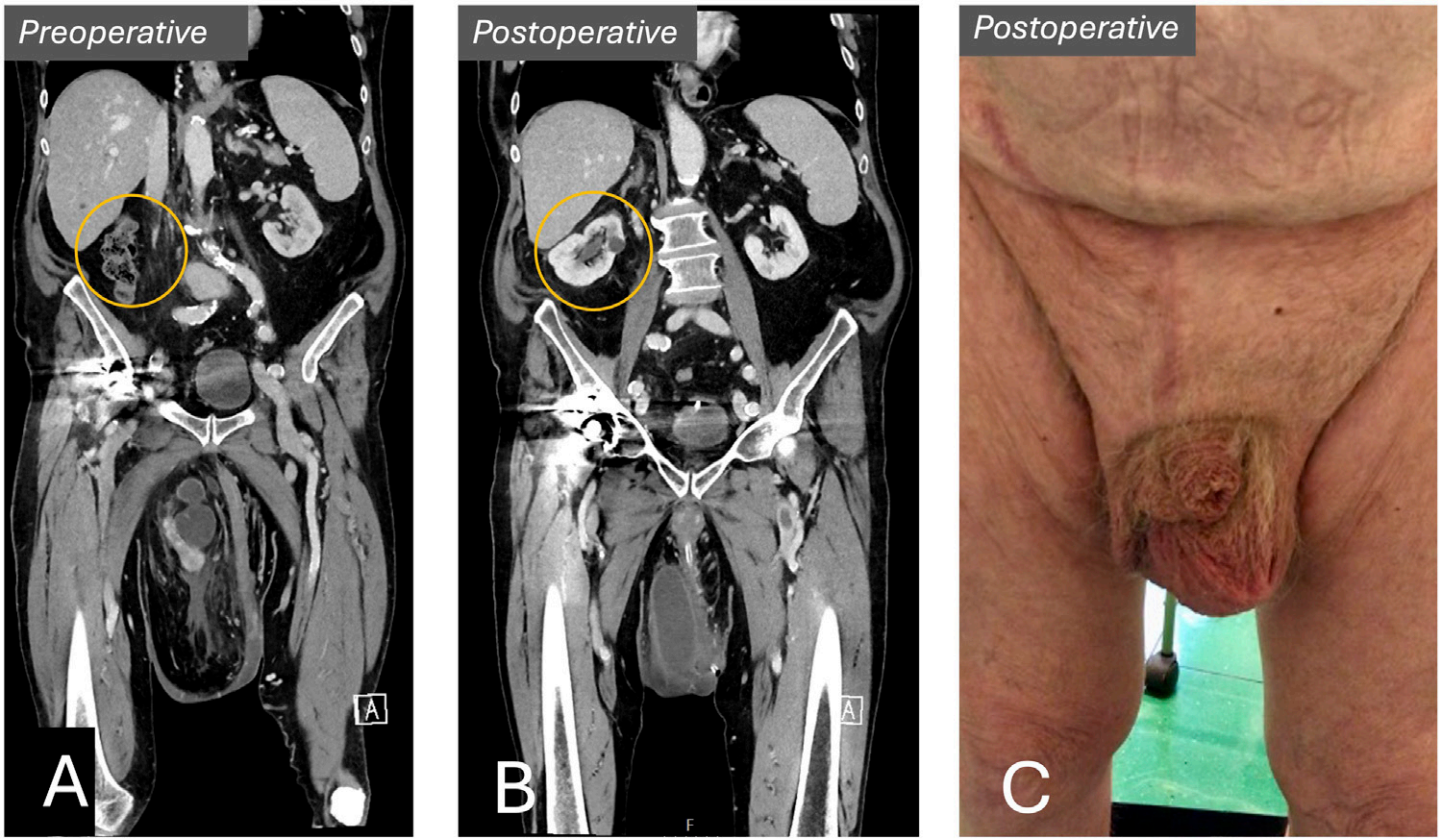
Preoperative vs. postoperative. **(A)** Preoperative CT-scan showing kidney within scrotum. **(B)** Postoperative CT-scan showing right kidney in its normal anatomical position within the right flank. **(C)** Postoperative result.

The postoperative course was uneventful. The patient was mobilized on the day of surgery with low pain levels. Kidney function was monitored daily for evidence of possible malrotation of the kidney or “kinking” of the ureter. The patient was discharged from the hospital on the fourth postoperative day. A 6-week postoperative CT-scan showed quite surprisingly that the right kidney had returned to its normal anatomical position within the right flank (Figure 4.). Both CT and renography showed an elongated hydroureter, but good passage of contrast and equal distribution of renal function. A scrotal seroma was drained at the 6-week and 12-week postoperative control. There were no signs of infection. The patient scored 0 points on the *Carolina´s Comfort Score* 6 weeks postoperatively [7]. Moreover, he reported prolonged beneficial effects of the preoperative prehabilitation program, experiencing increased muscle strength and better mobility. The seroma has resolved completely at the 6-month follow up. (Figure 4C). The patient had started engaging in daily one-hour walks and expressed the feeling like he had begun a new life.

## Discussion

This case report describes an uncommon, incarcerated giant sliding hernia with kidney involvement. Literature revealed nine documented cases of scrotal hernias involving the kidney [8–16]. Four cases underwent surgery. [8, 13, 14, 16]. One case presented with acute incarceration and renal failure [13]. Laparotomy and renal fixation were performed in two patients, one fatal due to necrotizing fasciitis [8, 14]. In one case, the surgical procedure was not described [12, 13]. In the fourth case, a trans-inguinal approach without kidney fixation was performed, similar to our case [16]. This last case required retrograde ureter stenting due to “kinking of the ureter” leading to obstruction. None of the cases presented a detailed operative description. Our paper is unique with a complete preoperative, surgical and postoperative management. Also, a12-month follow-up are recorded and discussed in detail. The characteristics of this case and the learning points will be discussed below.

Our giant scrotal hernia was classified as S3 (IR) under the amended European Hernia Society Classification of scrotal hernias (S1 = upper third thigh, S2 = middle third thigh, S3 = lower third thigh or below, IR = irreducible) [2]. Whether a hernia is irreducible or not is important, as this has implications for the type of surgical repair. Surgical repair of these massive scrotal hernias carries a higher risk of complications than non-scrotal inguinal hernias [2]. Our patient developed only a wound seroma, which may have been prevented by the placement of a drain during surgery.

The patient presented with acute sepsis due to an incarcerated hernia. A strangulated bowel might have been presumed. However, a preoperative CT scan revealed an incarcerated kidney. The importance of a CT scan, if possible, can therefore not be underestimated in cases of giant scrotal hernias as this also enables the assessment of “loss of domain.” Without imaging, the patient could have been subjected to an extremely difficult emergency hernia surgery with possible consequent significant complications.

Five essential steps in the pre-operative management of this patient are essential:

1. Sepsis treatment: This was effectively managed with drainage of the obstructed kidney and antibiotics.
2. Preoperative optimization: Beneficial effects of personalized training, smoking management, diets, mental support, hemoglobin and diabetes control are documented in multiple studies, but not systematically implemented within hernia surgery [6].
3. Augmentation of the abdominal cavity: Botox treatment in ventral- and complex inguinal hernias has been documented [17]. Augmentation of the abdominal cavity might prevent abdominal compartment syndrome. Progressive Preoperative Pneumoperitoneum (PPP) technique and Botox injections into the oblique muscles are the two available options. We opted for Botox, commonly used in our hospital. It is worth noting that in the experience of Operation Hernia, repair of massive scrotal hernias almost never results in compartment syndrome.
4. Consent for Orchidectomy: Orchidectomy is avoidable but may be necessary. It may facilitate a difficult dissection of a densely adherent sac, and might be desirable to reconstruct a destroyed posterior wall. In our case the peritoneal sac was difficult to identify. Complete closure of the internal ring ensured a satisfactory “closure of the sac,” an essential step in inguinal hernia surgery.
5. Skill of surgeon: Our surgeons were able to handle all steps in this giant hernia due to valuable experience acquired in LMIC through Operation Hernia. Patients with giant scrotal hernia should be referred to specialist centres and handled by surgeons with the appropriate training and experience.

Given the complexity of massive scrotal hernias, a structured surgical approach is essential. Some of the key steps of the operation are discussed in more detail: 1) reduction of the scrotal hernia, 2) skin incision, and 3) management of the scrotal skin. Successfully reducing the hernia transforms a challenging anatomical scenario into a more recognizable one. This should be performed in stages, as demonstrated in this case:

1. Preoperative reduction by patient or by surgeon before anaesthesia.
2. After establishment of general or spinal anaesthesia.
3. After incision of the External Oblique fascia into the external ring.
4. After incision of the internal ring (Internal Oblique muscle as described in the text).

However, reducing a tender, strangulated scrotal hernia is inadvisable, as necrotic bowel may be reduced into the abdomen. The skin incision was extended to the scrotum for better access to the external ring [4]. Based on our experience, removing excess scrotal skin is unnecessary, as it increases the risk of complications and typically contracts naturally after surgery.

We consider our case to be extreme nephroptosis (“floating kidney”) in which an orthotopic kidney and retroperitoneum had gradually descended into the scrotum over several years. Other authors have described incarcerated pelvic kidneys [11, 15, 16]. The distinguishing features include (1) the anatomy of the renal vessels, which in our case originated at the typical level from the aorta just below the superior mesenteric artery, and (2) the length of the ureters, being elongated in our case, as opposed to them being shortened as in pelvic kidneys. The kidney was not fixed and no ureter stents were placed, but we were prepared to perform retrograde or antegrade stenting if necessary.

## Data Availability

The original contributions presented in the study are included in the article/supplementary material, further inquiries can be directed to the corresponding author.
